# Molecular network of the oil palm root response to aluminum stress

**DOI:** 10.1186/s12870-023-04354-0

**Published:** 2023-06-30

**Authors:** Fernan Santiago Mejia-Alvarado, David Botero-Rozo, Leonardo Araque, Cristihian Bayona, Mariana Herrera-Corzo, Carmenza Montoya, Iván Ayala-Díaz, Hernán Mauricio Romero

**Affiliations:** 1 Colombian Oil Palm Research Center - Cenipalma, Oil Palm Biology, and Breeding Research Program, Bogotá, 11121 Colombia; 2 Department of Biology, Universidad Nacional de Colombia, Bogotá, 11132 Colombia

**Keywords:** Abiotic stress, Differential expression analysis, Gene co-expression network, *Elaeis guineensis*, STOP1

## Abstract

**Background:**

The solubilization of aluminum ions (Al^3+^) that results from soil acidity (pH < 5.5) is a limiting factor in oil palm yield. Al can be uptaken by the plant roots affecting DNA replication and cell division and triggering root morphological alterations, nutrient and water deprivation. In different oil palm-producing countries, oil palm is planted in acidic soils, representing a challenge for achieving high productivity. Several studies have reported the morphological, physiological, and biochemical oil palm mechanisms in response to Al-stress. However, the molecular mechanisms are just partially understood.

**Results:**

Differential gene expression and network analysis of four contrasting oil palm genotypes (IRHO 7001, CTR 3-0-12, CR 10-0-2, and CD 19 − 12) exposed to Al-stress helped to identify a set of genes and modules involved in oil palm early response to the metal. Networks including the ABA-independent transcription factors DREB1F and NAC and the calcium sensor *Calmodulin-like* (CML) that could induce the expression of internal detoxifying enzymes GRXC1, PER15, ROMT, ZSS1, BBI, and HS1 against Al-stress were identified. Also, some gene networks pinpoint the role of secondary metabolites like polyphenols, sesquiterpenoids, and antimicrobial components in reducing oxidative stress in oil palm seedlings. STOP1 expression could be the first step of the induction of common Al-response genes as an external detoxification mechanism mediated by ABA-dependent pathways.

**Conclusions:**

Twelve hub genes were validated in this study, supporting the reliability of the experimental design and network analysis. Differential expression analysis and systems biology approaches provide a better understanding of the molecular network mechanisms of the response to aluminum stress in oil palm roots. These findings settled a basis for further functional characterization of candidate genes associated with Al-stress in oil palm.

**Supplementary Information:**

The online version contains supplementary material available at 10.1186/s12870-023-04354-0.

## Background

Different types of abiotic stresses are considered limiting factors in crop yield [1]. Natural or anthropogenic events cause soil acidity (pH < 5.5), representing a significant crop limitation. About 30% of the total cropland in the world is considered acidic [2]. In South America, the pH of soils tends to be extremely low (pH < 3.5), and Colombia presents a broad diversity of soils where approximately 85% have pH values below 5.5 [3]. Soil acidification is not considered a class of abiotic stress. However, it enables a significant increment of metal ions, especially aluminum (Al^3+^) ions, in the rhizosphere. Root plants uptake Al^3+^ species which competes chemically with other nutrients [4]. Al ions affect DNA and cell division, triggering root morphological alterations and water uptake inhibition; these disorders affect the crop yield [5, 6].

Al effects have been documented in several plants. Environmental, physicochemical, and genotype conditions determine if Al species are toxic or beneficial for the plant [2, 7, 8]. In oil palm, some studies have described Al’s morphological, physiological, and biochemical effects in African oil palm (*Elaeis guineensis* Jacq.), interspecific OxG (*E. oleifera* x *E. guineensis*) hybrids, and clones [9–12]. Other studies have been focused on identifying different soil amendments techniques and the relation of Al with the nutrient content and even with some biotic stresses [13–15].

Some strategies have been implemented to mitigate soil acidity and Al-ion phytotoxicity. For instance, calcareous by-products and lime reduce the content of Al in soils and increase pH. Boron, calcium silicate, and phosphorus have been used and gradually accepted by farmers as exogenous fertilizers and soil conditioners [16–19]. However, these practices are laborious, expensive, and, in some cases, low efficient. Another alternative to mitigate Al as a long-term and durable solution is the implementation of plant breeding strategies through genetic resource exploration, tolerance mechanics, and genomic tools for developing Al-tolerant plant materials [3, 10–13].

Oil palm is a perennial tree and the principal source of vegetable oil worldwide. *E. guineensis* is native to central and west Africa and cultivated in humid tropical regions. Indonesia, Malaysia, and Thailand are the top-three oil palm producers, whereas Colombia is the largest crude palm oil producer in Latin America [20]. According to the National Federation of Oil Palm Growers of Colombia (FEDEPALMA), around 595,723 ha of arable land are cultivated with oil palm in Colombia (African and OxG hybrid) distributed in four geographic zones: North, Central, East, and South-west; all these zones present high acidic soils, except the north region [11]. In this sense, oil palm cultivations in acidic soils represent a challenge for smallholders and large-scale producers due to limiting conditions.

Some studies have analyzed root architecture, biomass, length, and mineral element absorption to identify oil palm Al-tolerant and Al-sensitive genotypes. Also, photosynthetic characteristics, reduction in fruit bunch yield, oil extraction potential, sugar, and organic acid content have been described in response to Al. These results allowed us to identify oil palm-specific morphological, physiological, and biochemical responses to overcome Al-stress. Nevertheless, the molecular mechanisms are still incomplete or partially understood.

RNA-Seq is one of the most used techniques to examine gene expression profiles. Some studies in Al-stressed plants enable the discovery of gene alterations associated with membrane transporters, signal transduction, transcription factors, oxidative stress, cytoskeletal dynamics, energy, and metabolism [2, 19, 21].

Using gene co-expression networks constitutes an interesting approach to complement differential gene expression analysis. Co-expression network analysis is a computational and mathematical approach to interpreting a list of differentially expressed genes. Network analysis may be used to explore genes, proteins, metabolites, pathways, or interactions between them in a temporal and spatial co-regulation manner to understand biological phenomena [22, 23]. Differentially expressed genes can be connected and represented in a mathematical network based on their co-expression profiles. Then, across several measurements, such as centrality, degree, and modularity, the genes and their connections can be characterized to infer biological functions and connections. For example, “guilt-by-association” is an application used to identify and predict the gene function of unknown genes based on their network partners. Also, the network’s gene connections may represent regulators-target relationships or structural genes belonging to a specific metabolic pathway [23]. Thus, this project aimed to identify genes, networks, and transcription factors involved in oil palm response to Al using transcriptomic analysis and systems biology. We performed differential gene expression and network analysis of four contrasting oil palm genotypes under exposure to Al-stress. In addition, a subset of 12 differential expressed and network-relevant genes were chosen for RT-qPCR validation. Our results set a theoretical basis for oil palm molecular response to Al-stress and provide essential information for future genetic improvement and cultivar development.

## Results

### Growth traits and root architecture

**Fig. 1:**
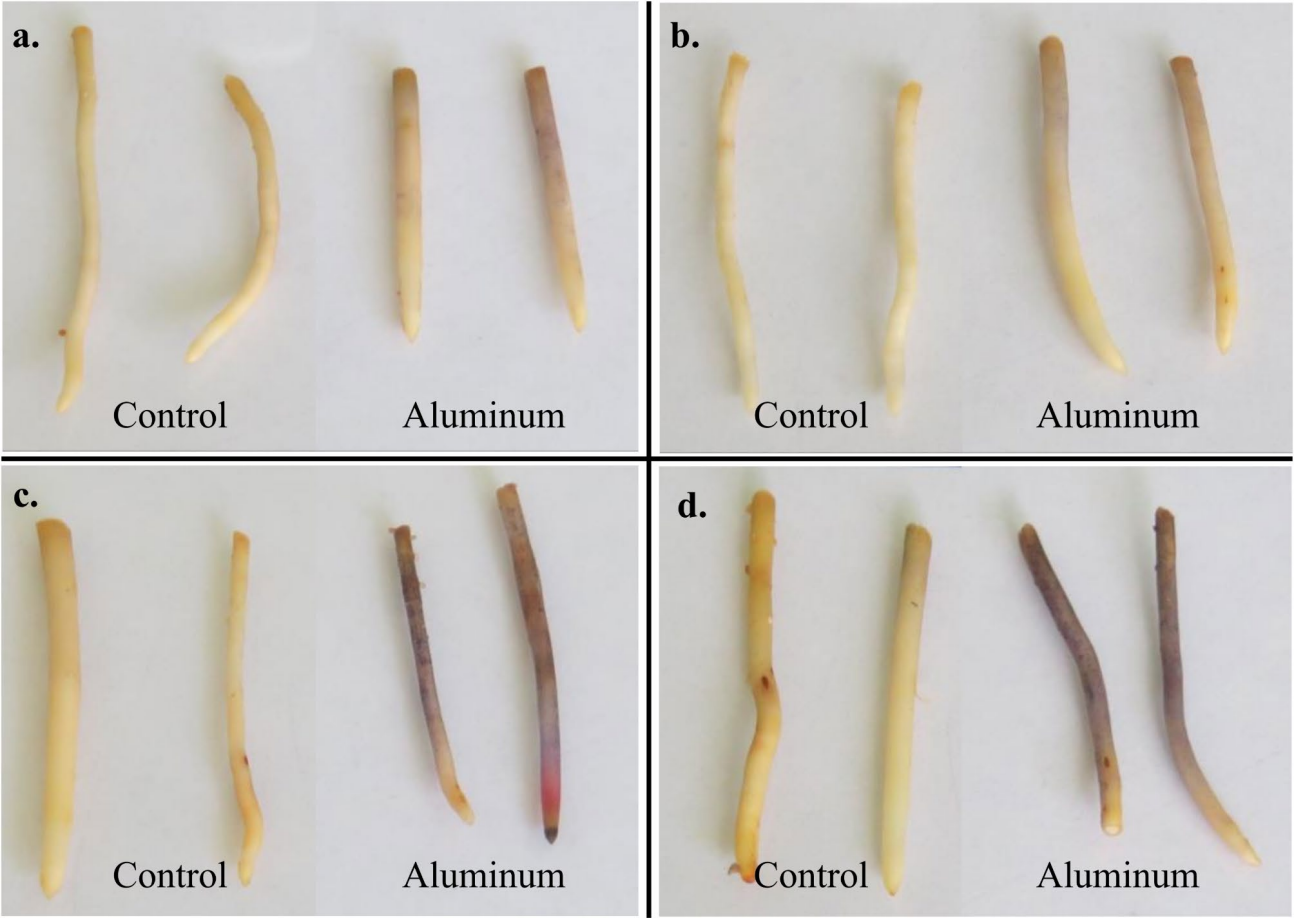
Hematoxylin staining of primary roots. (**a**) IRHO 7001, (**b**) CTR 3-0-12, (**c**) CR 10-0-2, and (**d**) CD 19 − 12, subjected to aluminum stress. A higher intensity and proportion of purple or blue colors represent a more substantial aluminum accumulation in the root apex and elongation zone

Hematoxylin staining in roots showed a significant accumulation (*p-value* < 0,05) among Al-treatments and controls in all genotypes (Additional file 1 supplementary Table 1). However, IRHO 7001 and CTR 3-0-12 show a small Al accumulation in primary roots (Fig. 1). Despite the Al-treatment decreasing the root length and the architecture of the root under Al-stress in all genotypes, no significant differences in the morphological characteristics were found (*p-value* = 0.459). The Al treatment increases the root tip number in IRHO 7001 and CTR 3-0-12.

### Differential gene expression and GO enrichment analysis

**Fig. 2:**
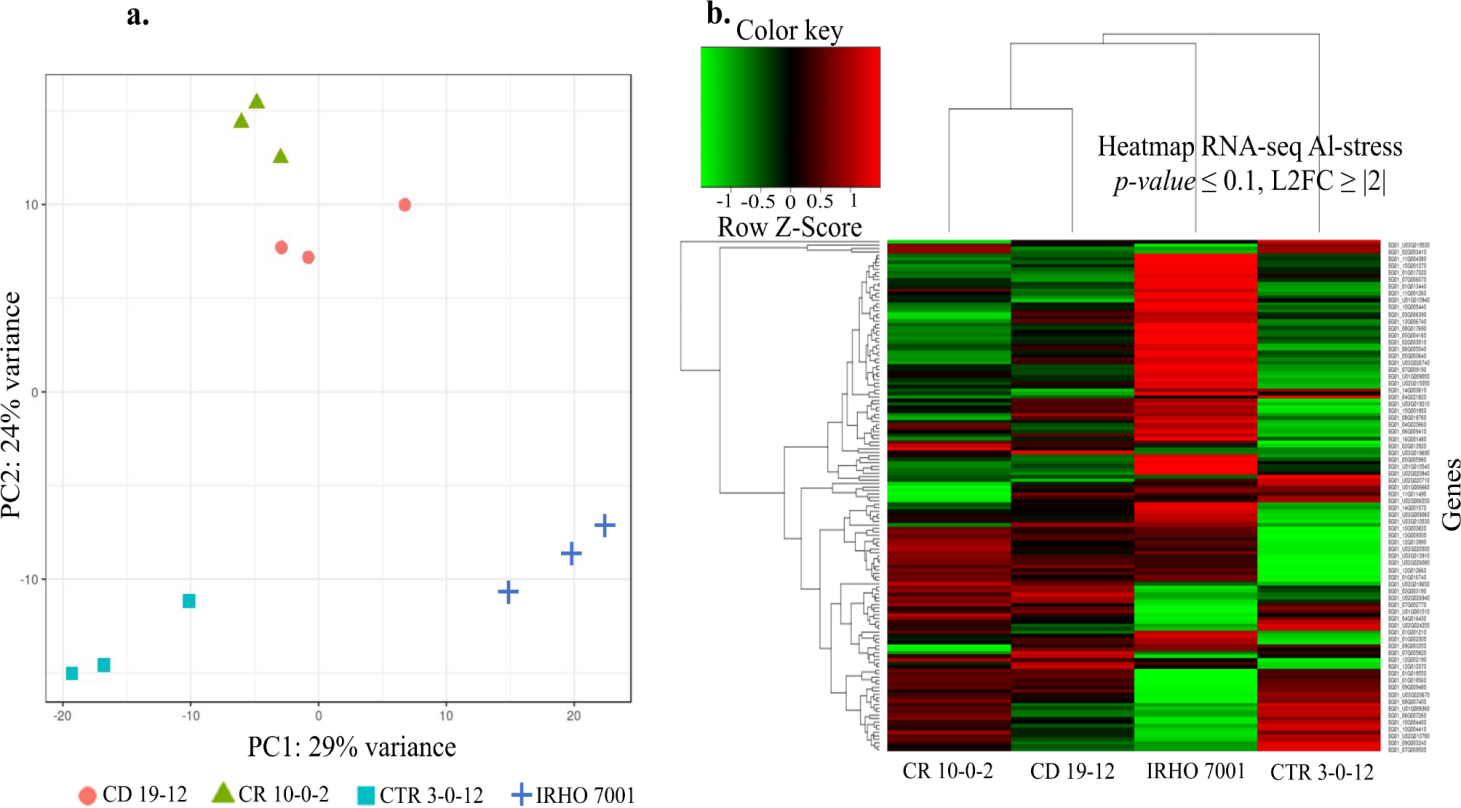
PCA and Expression profiles of oil palm genotypes under Al-stress. (**a**) Principal component analysis (PCA) of the RNA-seq samples among Al-tolerant and Al-sensitive genotypes in oil palm. (**b**) Expression profiles of differentially expressed genes in four oil palm genotypes under Al-stress

The RNA-Seq generated 148.9 Gb of raw information from 24 samples. The count range was 103,167,310 and 160,888,822 paired reads per sample (Additional file 1 supplementary Table 2). DESeq2 algorithm identified 148 differentially expressed genes (*p-value* ≤ 0.1 and LFC |2|). According to the PCA (Fig. 2a) and the heatmap (Fig. 2b), CR10-0-2 and CD 19 − 12 genotypes were differentiated from IRHO 7001 and CTR 3-0-12 genotypes. Interestingly, the genotypes IRHO 7001 and CTR 3-0-12 were separated between them. The expression profiles showed differences between IRHO 7001 and CTR 3-0-12, suggesting different response mechanisms to Al-stress.

IRHO 7001 was enriched in several biological processes: (1) carbohydrate and amino acid metabolism related to membrane biosynthesis; (2) oxidative stress related to the regulation of transcription, defense response, and signal transduction; (3) transmembrane metal transport. Other essential processes enriched in this genotype were iron ion homeostasis, related to regulating photoperiodism and flowering, and pigment biosynthesis and photosynthesis. According to apoplast transport activity, the cellular components were the extracellular region, cell wall, and membrane.

CTR 3-0-12 was enriched in biological processes of protein ubiquitination and phosphorylation related to lipid metabolism, metal, and calcium transport, regulation of transcription, response to oxidative stress, photosynthesis, light harvesting, and phosphatidylinositol dephosphorylation. Additionally, in molecular function, heme binding was enriched. At cellular components, again, membrane and photosystem I were enriched.

CD 19 − 12 was enriched in biological membrane transport processes, especially in iron ion and cation and cellular iron ion homeostasis. Complementary, at molecular function, ferric iron was related to sulfur, ATP, and DNA binding; oxidoreductase activity was related to chlorophyllide A. Here, only the membrane was enriched.

CR10-0-2 was enriched in the biological process of transmembrane and iron ion transport, iron ion homeostasis related to the regulation of transcription, sucrose metabolism, and protein phosphorylation. Only an integral component of the membrane was enriched.

### Gene co-expression network

**Fig. 3:**
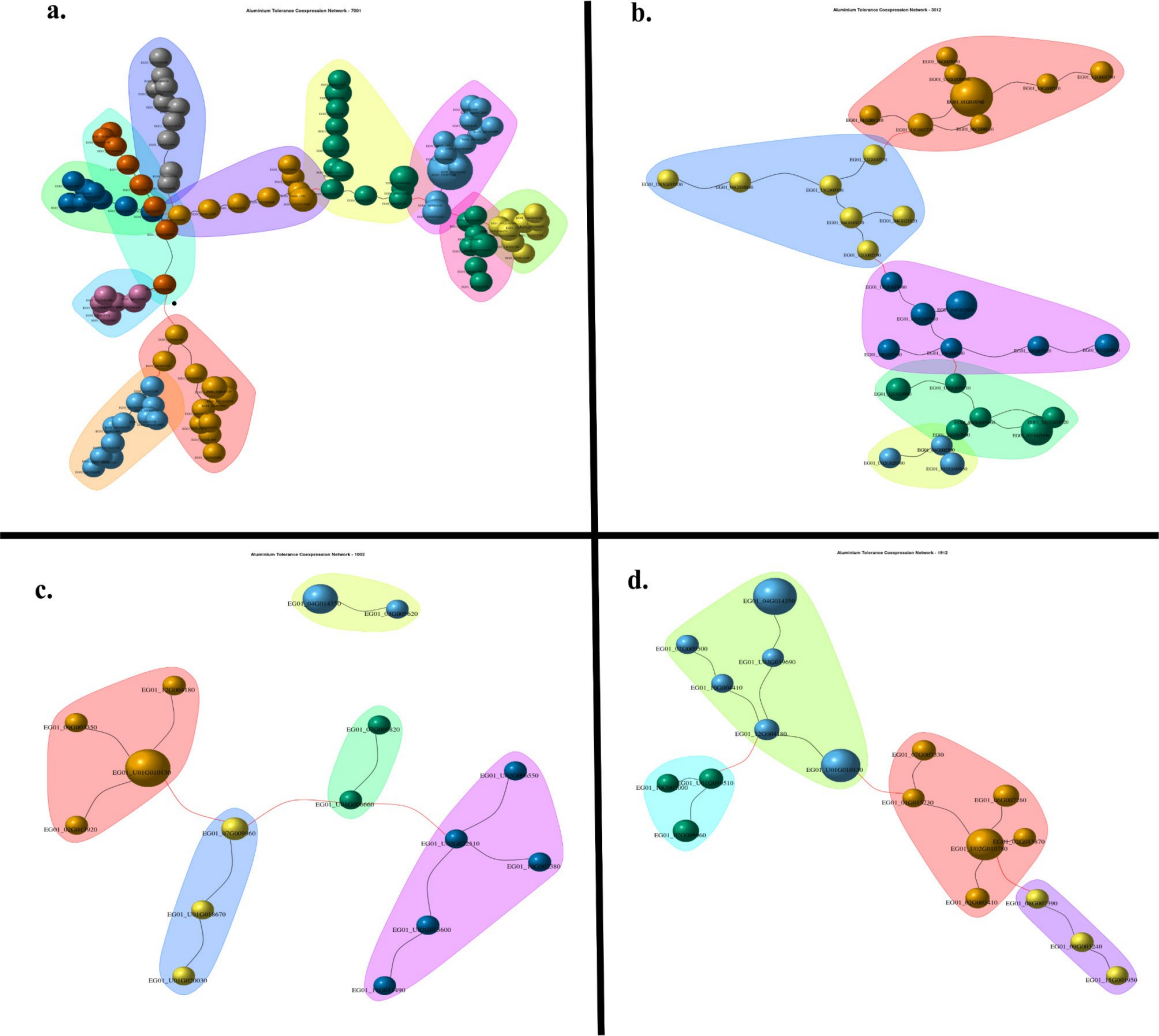
Gene co-expression network for each cultivar. (**a**) IRHO 7001, (**b**) CTR 3-0-12, (**c**) CR 10-0-2, and (**d**) CD 19 − 12. Each node (sphere or bead-like shape) represents a gene, and groups of nodes highlighted with the same color indicate a module of genes. Black edges represent a direct correlation between genes and the red lines’ inverse correlation. The size of the nodes is proportional to the mean level expression of the gene represented by the node. The *igraph* R package was used to construct the cultivar-specific co-expression networks under Al and control treatment

The general co-expression network for all genotypes was constructed based on 148 differentially expressed genes with 93 nodes (Additional file 2 supplementary Table 2). This network presented the higher modularity coefficient and lowest centralization measurements, showing 15 modules and 34 HUB genes (Additional file 2 supplementary Fig. 1). The *Bowman-Birk type trypsin inhibitor-like* presented the highest HUB score in this network, suggesting an essential role in this model (Additional file 2 supplementary Table 3). The largest genotype-specific co-expression network was for IRHO 7001, and it was constructed with 104 differential expressed genes and an exact number of nodes. This co-expression network presented the highest modularity, diameter, centralization betweenness, and average path length among the specific networks and had the lowest edge density, centralization degree, and closeness. For this network, 27 HUB genes were identified and grouped in 11 modules (Fig. 3a). The under-expressed *Pleiotropic drug resistance protein 3-like* (PDR3) gene was classified as the primary HUB gene for this network (Additional file 2 supplementary Table 4).

The CTR 3-0-12 genotype co-expression network was constructed using 34 differential expressed genes (31 nodes) and followed in the rank network metrics to IRHO 7001. However, there were considerable differences among several metrics: modularity, density, degree, and path length. In this network, 16 genes had high HUB scores (Fig. 3b). The *Dehydration-responsive element-binding protein 1 F-like* (DREB1F) was under-expressed and predicted as a HUB gene in this network (Additional file 2 supplementary Table 5). The CR10-0-2 and CD 19 − 12 gene co-expression networks (Fig. 3c and d) showed the smallest networks. They ranked at the bottom of the network metrics compared with IRHO 7001 and CTR 3-0-12 networks (Additional file 2 supplementary Table 2). Co-expression networks of CR 10-0-2 and CD 19 − 12 genotypes were constructed using 16 and 18 differentially expressed genes, respectively (same number of nodes); 14 and 7 genes were identified as HUB genes. A *Doubtful hypothetical protein* was predicted as the HUB gene for the CD 19 − 12 network (Additional file 2 supplementary Table 6), while the *transcription factor bHLH100-like* (bHLH100) gene was for the CR 10-0-2 network (Additional file 2 supplementary Table 7).

### RT-qPCR validation

**Fig. 4:**
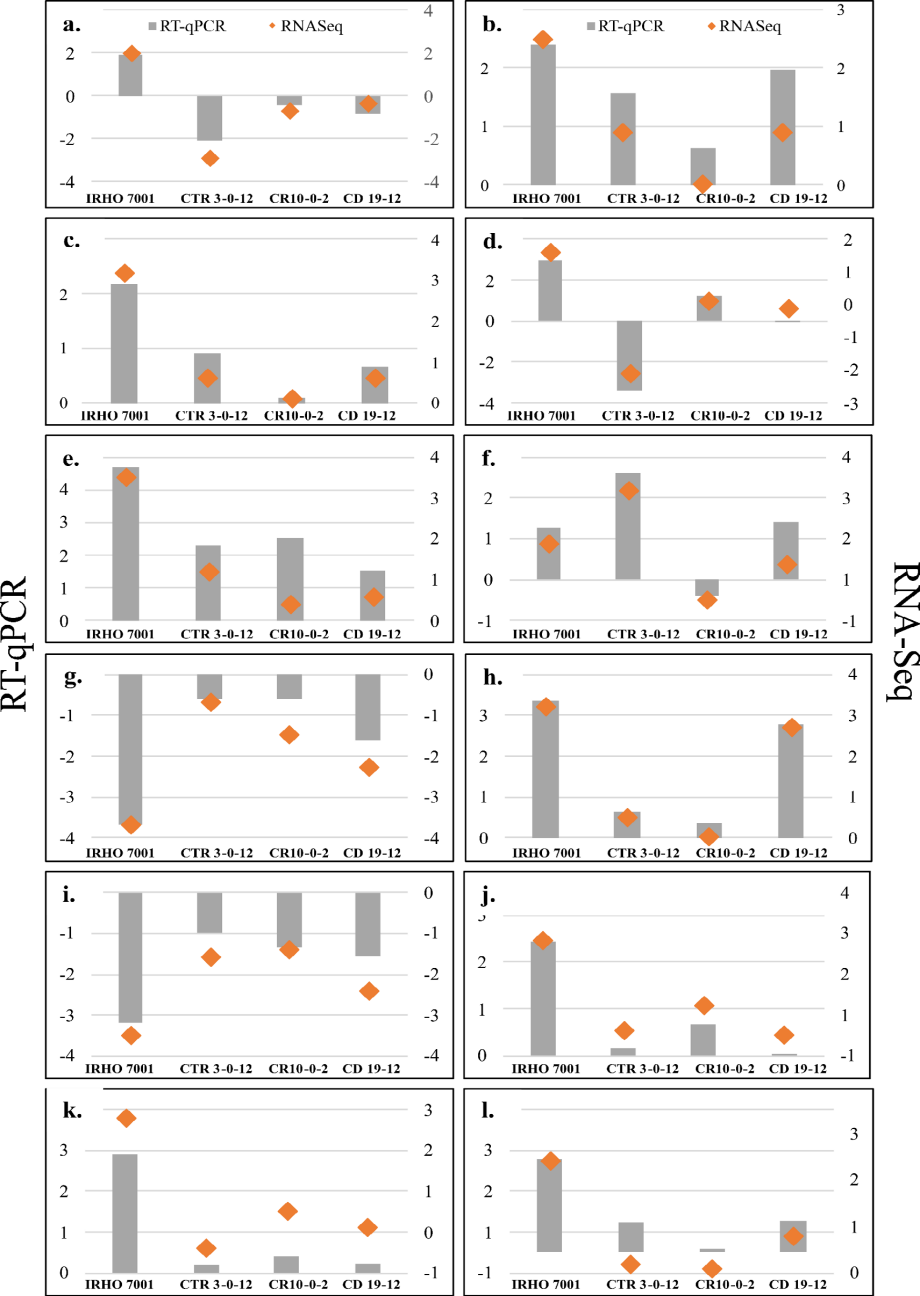
Genes validated by RT-qPCR. (**a**) Trans-resveratrol di-O-methyltransferase-like (ROMT); (**b**) NAC domain-containing protein 72 (NAC); (**c**) protein PHLOEM PROTEIN 2 (PP2); (**d**) Dehydration-responsive element-binding protein 1 F (DREB1F); (**e**) Late embryogenesis abundant protein D-34 (LEA34); (**f**) Stress-response A B barrel domain-containing protein At5g22580; (**g**) transcription factor bHLH100 (bHLH); (**h**) vacuolar iron transporter 1.1 (VIT1.1); (**i**) Pleiotropic drug resistance protein 3-like (PDR3); (**j**) 9-cis-epoxy carotenoid dioxygenase (NCED); (**k**) UPF0481 protein At3g47200; (**l**) Phytoene synthase 2 (PSY2). Grey bars indicate the value expression obtained by RT-qPCR. Orange diamonds indicate the RNA-Seq value

To verify RNA-Seq data, the selection of 12 genes for RT-qPCR validation was based on the following parameters: (1) HUB score and connectivity with other genes into the gene co-expression network, (2) contrasting expression among genotypes, and (3) previously reports in the literature as significant in Al-stress tolerance (Fig. 4). We did not observe significant differences in the expression of reference gene NADH among treatments and genotypes (Kruskal-Wallis Test *p-value* = 0.1449). This indicates that NADH presented a stable expression among genotypes and treatments and was appropriately used as a reference gene. The expression of every single gene was evaluated for each genotype. A high association (r = 0.94) was found between RNA-Seq vs. RT-qPCR data (Additional file 2 supplementary Fig. 2). This result suggests that the transcriptomic data are reliable.

## Discussion

A transcriptome and gene co-expression network analysis was conducted to identify differentially expressed genes and biological and molecular functions involved in oil palm response to Al-stress. Gene expression is an essential process necessary to adapt the cell to diverse stimuli, and these stimuli are sensed and translated by the cell, triggering tolerant or sensitive responses [24]. In this study, co-expression networks were constructed from 148 differentially expressed genes to identify genes or modules of genes related to a biological function involved in Al-stress response. The general co-expression network predicted the overexpressed *Bowman-Birk type trypsin inhibitor-like* (BBI) as a HUB gene. BBIs are a natural protease inhibitor family reported in several biological-defensive functions in plants and animals [25, 26]. In oil palm, BBI has yet to be studied. However, James and collaborators [27] aligned BBI sequences using BLASTp with several plant sequences, including eight from *E. guineensis.* They found a sequence similarity with pineapple (*Ananas comosus*) in a monocot clade (bootstrap support ≥ 73.5%).

In CR 10-0-2 and CD 19 − 12 genotypes, four response genes were overexpressed; *ferritin-4 2 C chloroplastic* (FER4) and *oligopeptide transporter 3-like* (OPT) were the most relevant genes, respectively. Ferritin is a multimeric complex whose primary function is the source reallocation of iron under deficiency conditions [28, 29]. Low mineral absorption is one of the symptoms caused by Al stress in plants; thus, Al could interfere with iron metabolism activating FER4 and *vacuolar iron transporters* (VIT) involved in vacuolar Al sequestration. Meanwhile, OPT overexpression could be caused by changes in the membrane induced by Al-stress, growth regulat*ory* induction, or transportation process [7].

In general, there were different topologies among co-expression networks. IRHO 7001 presented a more extensive, modular, and interconnected (betweenness) co-expression network in contrast with the other networks. The genes with the highest HUB score were under-expressed in IRHO 7001 and CTR 3-0-12 genotypes. Here, PDR3 and DREB1F could be negatively regulated by other response genes. The PDR3 gene codifies an ABC transporter (ATP-binding cassette) and has been reported as a general defense protein [30]; this gene is induced by growth regulators like methyl jasmonate (MeJA) and salicylic acid (SA), even for some mineral deficiencies like iron deficiency [31]. Related to CTR 3-0-12 network, DREB-type factors are members of the AP2/ERF family (*APETALA2/Ethylene Responsive Element Binding Factor*); this family is composed of numerous essential transcription factors (TFs) known to play an important role in gene expression regulation in response to abiotic stresses via abscisic acid (ABA) independent and ABA-dependent pathways [32, 33]. Wang and collaborators [34] studied the DREB1F gene in *Arabidopsis* and rice; they found DREB1F was constitutively expressed in many tissues, and its overexpression confers tolerance to several abiotic stress (high salinity, drought, and low temperatures), but not by oxidative stress, wounding, and pathogen attack. In oil palm, Azzeme and collaborators [35] studied the contribution of DREB1 in the stress signaling pathway in seedlings. They performed an ectopic expression of EgDREB1 in T1 transgenic tomato seedlings, improving the expression of several antioxidant genes in response to drought stress.

Several studies have shown that external (apoplast) and internal (symplast) detoxification strategies constitute mechanisms for Al tolerance response. External detoxification involves modifications of the plasma membrane, exudation of organic acids, and change of the pH in the rhizosphere. Internal detoxification includes chelation in the cytosol by secondary metabolites, compartmentation in the vacuole, and high enzymatic detoxification activity [36, 37].

Our results did not show significant changes in root length and architecture. However, we observed an increment in the number of tips induced by Al in IRHO 7001 and CTR 3-0-12 genotypes (Additional file 1 supplementary Table 1). The overexpression of various genes related to the cell wall reorganization process, transcriptions factor, phytohormones precursors, ABC-transporters, and ionic sensors, helps to increase the numbers of tips in roots, suggesting an internal and early mechanism of tolerance response against Al-stress in IRHO 7001 and CTR 3-0-12 genotypes. The root area increment might allow a significant quantity of secondary metabolite exudation or production of detoxification enzymes to overcome Al-stress. These findings were previously reported by Rivera-Méndez and collaborators [11], where damage in interspecific OxG hybrid root growth induced by Al was related to alterations in the redox state, cell division, and elongation.

**Fig. 5:**
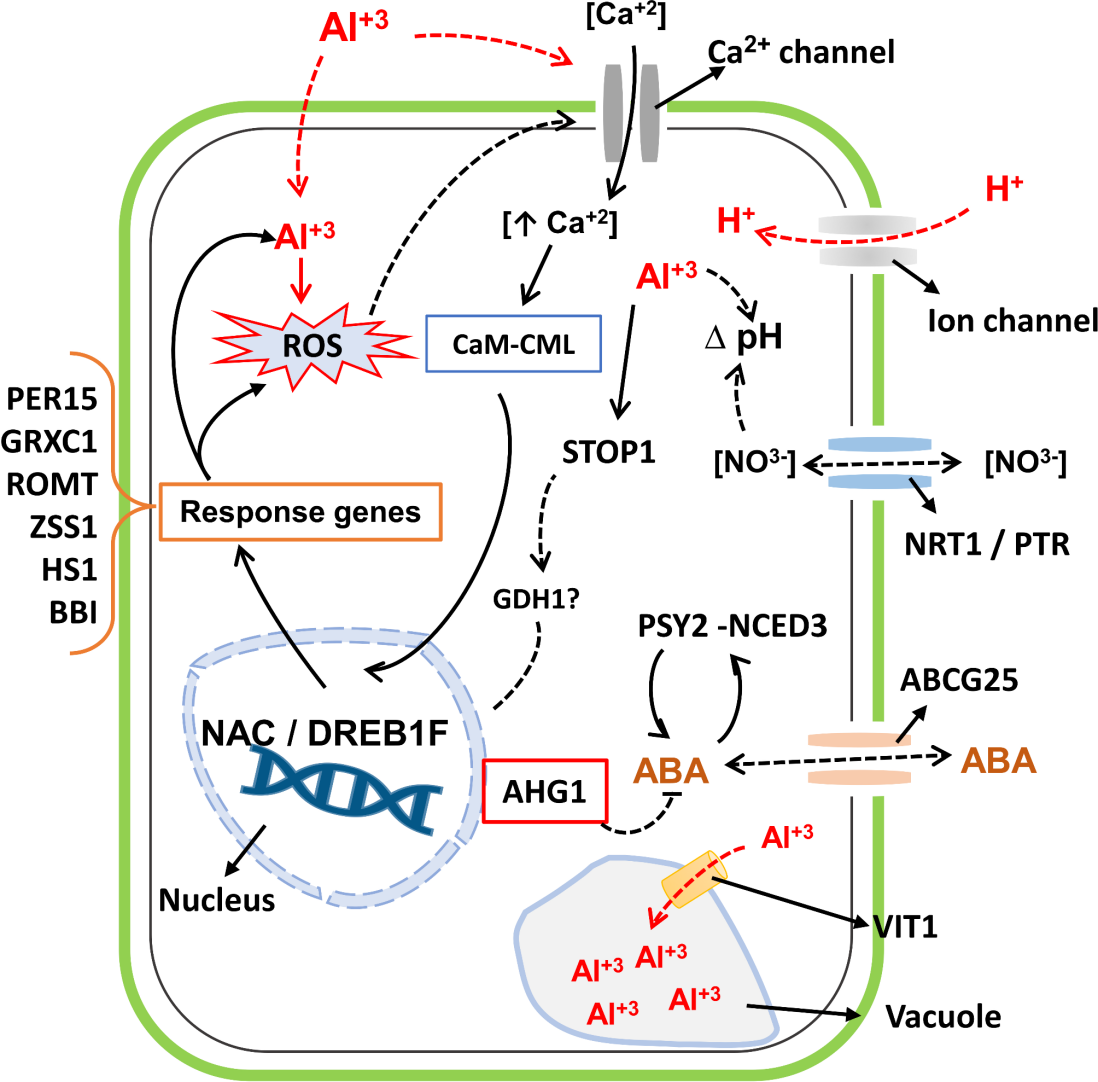
Possible gene response to Al-stress in oil palm. The stimuli of Al-stress and ROS might induce the expression of transcription factors such as NAC and DREB1F and calcium sensor *Calmodulin-like* (CML), which regulate detoxification enzymes such as GRXC1, PER15, ROMT, ZSS1, BBI, HS1, and ABA inductor against Al-stress in oil palm. STOP1 and ABA-mediated response might induce an external detoxification mechanism. Black straight lines represent the direct association between genes, and dashed lines are hypothetical associations based on literature

We hypothesize that once the roots sense Al, it crosses the cell wall and the plasma membrane (through membrane transporters), affecting the membrane potential of root cells, root elongation, nutrients, and water uptake [38, 39]. Al interacts with multiple root cell sites in the cytoplasm, and its affinity represents an important toxicity factor. This interaction increases the Reactive Oxygen Species (ROS) concentration, causing irreversible cell damage (Fig. 5). However, plants integrate ROS as signaling molecules or secondary messengers under limiting conditions. ROS could regulate other secondary messengers (i.e., cytosolic Ca^2+^), activating pumps and carrier proteins that maintain homeostasis in the cell [40, 41]. Thus, the increment of ROS, Ca^2+^, and Al in the cytosol could change the ionic homeostasis, activating internal mechanisms and transcription factors downstream to deal with Al stress. Also, these secondary messengers can modulate the pH in the cytosol through activating Mg^2+^, NO^3−,^ and peptide transporters [42]. Indeed, oil palm might activate calcium sensors as *Calmodulin-like* (CML) proteins or ABA-independent transcription factors such as DREB1F and NAC (acronym of NAM, *no apical meristem*, ATAF1/2, *Arabidopsis* and CUC2 *cup-shaped cotyledon*) to encode detoxification enzymes. One of these enzymes, *glutaredoxin-C1-like* (GRXC1), is involved in cellular proteome retrieval. Another one, *peroxidase 15-like* (PER15), can reduce hydrogen peroxide (H_2_O_2_). GRXC1 and PER15 are involved in the biogenesis of cellular Fe/S proteins and restore osmotic homeostasis in the cell [43, 44].

DREB1F, NAC, and CML might activate other functional genes reported in the biosynthesis of secondary metabolites that confer Al tolerance and reduce oxidative stress. Among those, the *trans-resveratrol di-O-methyltransferase-like* (ROMT) has been reported as a precursor in the biosynthesis of polyphenols in response to the Al-stress in grapevine [45].

Another defense-associated gene in the sesquiterpenoid biosynthetic pathway is the rhizome *alpha-humulene synthase-like* (ZSS1) [46]. Also, the *Stress-response A/B barrel domain-containing protein At5g22580* (homolog of *Stress-response A/B barrel domain-containing protein* HS1) has been reported in the biosynthesis of antimicrobial components [47]. Interestingly, we found overexpressed genes reported in drought transcriptome response. Thus, oil palm might perceive Al stimuli as a water uptake issue, activating the transcription factors DREB1F and NAC as internal response-based mechanisms on ROS and Ca^2+^ signaling in ABA-independent pathways.

The constant stimulus of Al^3+^ and H^+^ in the cytoplasm possibly activates other transcription factors involved in the Al tolerance response. The transcription factor *sensitive to proton rhizotoxicity 1* (STOP1) is a zinc finger TF which, together with *protein sensitive to proton rhizotoxicity 2* (STOP2), *glutamate-dehydrogenase1 and 2* (GDH1 or GDH2), or *Aluminum-activated malate transporter 1* (ALMT), plays a vital role in H^+^ and aluminum ion Al^3+^ tolerance [48–51]. Here, the transcriptomic analysis did not show STOP2, GDH1, and GDH2 gene expression; instead, ALMT and MATE (*multidrug and toxic compound extrusion*) were differentially expressed but not included in the co-expression network. Nevertheless, STOP1 was over-expressed, showing that under different limiting conditions, plants induce genes involved in the ABA biosynthesis and transportation [52].

In the ABA-dependent pathway, ABA signaling requires receptors, such as *pyrabactin resistance 1* (PYR1), *PYR1-like receptors* (PYL), and *regulatory components of ABA receptor 1* (RCAR1). Secondly, the ABA response is regulated by other components that act as ABA positive regulators, like *sucrose non-fermenting 1-related protein kinase 2* (SnRK2), and others that act as ABA negatively regulators, like *type 2 C protein phosphatase* (PP2C). Finally, *responsive elements like ABA-responsive transcription factors* (AREB) trigger the tolerance response [53]. We observed a profile expression related to early ABA biosynthesis signaling; we found an overexpressed *ABC transporter G family member 25-like* (ABCG25) gene, reported in ABA transportation from root xylem to cytosol. Possibly, when ABA enters the cell activates enzymes involved in carotenoid metabolism, the initial steps of ABA biosynthesis (*9-cis-epoxy carotenoid dioxygenase*, NCED), and other genes related to ABA biosynthesis like *Phytoene synthase 2* (PSY2) [53–55]. Thus, after three days under Al-treatment, oil palm could activate external detoxification mechanisms to counteract the Al-stress effects. However, the ABA negative regulator *probable protein phosphatase 2 C 75* (AHG1) was overexpressed. Perhaps, AHG1 is blocking the SnRK2 regulator, indicating no ABA-signaling in the early stages.

In summary, transcriptome analysis and systems biology provided a better understanding of the molecular network mechanisms in the oil palm root response to aluminum stress. The most expressed genes predicted by DESeq2 are involved in the biosynthesis of secondary metabolites, transcription factors, phytohormone biosynthesis precursors, and peptides and hormone transporters. Oil palm uses ROS secondary messengers, Ca^2+^, and Al, to induce ABA-independent pathways as an internal mechanism to reduce oxidative stress and induce Al-response genes.

Other researchers have reported the transcription factors DREB1 and NAC in abiotic stress responses under ABA-independent and ABA-dependent pathways. These transcription factors and calcium sensor *Calmodulin-like* (CML) might regulate detoxification enzymes such as GRXC1, PER15, ROMT, ZSS1, BBI, HS1, and ABA inductor against Al-stress in oil palm. The ROMT gene was reported in Al-response in grapes, while ZSS1, BBI, and HS1 genes have not been reported before in Al-stress response. The constant stimuli of Al-stress and ROS oxidative damage in a short time might induce an external mechanism based on ABA-dependent pathways. STOP1 expression could be the first step to the induction of ALMT and MATE genes as an external detoxification mechanism. ABA and Ca^2+^ signaling represent two independent signaling pathways, which could complement each other in the oil palm stress response. These findings provided a basis for further functional characterization of candidate genes associated with Al-stress in oil palm.

## Methods

### Location

The research was conducted in the Experimental Field Station “El Palmar de la Vizcaína,” located in the Department of Santander - Colombia (6° 59’ 3.4902”, -73° 42’ 19.6842”), at 140 m above sea level, with a relative humidity of 75%, the average temperature of 29 °C, and bimodal average annual rainfall of 3,200 mm.

### Plant material and experimental conditions

Sixty-four genotypes of the Cenipalma germplasm collection were subjected to high aluminum concentrations in hydroponic solutions under greenhouse conditions. Four contrasting *E. guineensis* (DxP) genotypes were selected from the collection based on the morphological and biochemical traits [11] length and root architecture, organic acid production, and hematoxylin stain were measured for each genotype under aluminum stress (data not shown). The four genotypes selected for this study were highly contrasting according to those diagnostic variables. IRHO 7001 and CTR 3-0-12 were aluminum tolerant, while CR 10-0-2 and CD 19 − 12 were aluminum-sensitive genotypes. The germinated seeds of the four selected oil palm genotypes (Additional file 2 supplementary Table 8) in phenological stage 004 were planted in sand beds up to phenological stage 102 with three leaves [56] in a half-strength Hoagland solution. The seedlings were kept for one month in a half-strength Hoagland solution for the acclimation period. The seedlings were held for three days in a half-strength Hoagland solution with 150 µM AlCl_3_ 6H_2_O (pH 4.2) for the Al stress treatment, according to Rivera-Méndez and collaborators [11]. The Al-free hydroponic solution was used as a control treatment for each genotype. A completely randomized block design with three replicates for each genotype was used.

### Growth traits and root architecture

The longest primary root was selected from each plant, placed in acrylic trays with distilled water, and scanned with an Epson Expression 10,000 XL scanner. Using the WinRHIZO software PRO V.3.5 (Regents Instruments Inc., Canada. https://regent.qc.ca/), the total number of tips and the total length of the primary, secondary, and tertiary roots were measured. In addition, following the method by Tang and collaborators [57], the roots were stained with hematoxylin, and the percentage of the stained area was determined using the WinRHIZO system.

### RNA isolation and library construction

Root samples were collected and homogenized with liquid nitrogen before RNA isolation. Total RNA was extracted from 100 mg of root tissue using InviTrap® Spin Plant RNA Mini Kit (Invitek Molecular, Berlin, Germany) according to the manufacturer’s protocol. Total RNA concentration and quality were examined using an Agilent 2100 Bioanalyzer Instrument (Santa Clara, CA, United States). RNA samples with RIN ≥ 7 were precipitated with 3 M sodium acetate and ethanol and sent to RNA-Seq library construction by Macrogen Europe BV (Amsterdam, the Netherlands). The libraries were constructed using a TruSeq Stranded Total RNA (Illumina, Inc. San Diego, CA, United States) and sequenced in the Illumina NovaSeq 6000 platform (Illumina, Inc. San Diego, CA, United States). The sequencing generated 101 bp paired reads.

### Differential gene expression and gene co-expression network construction

Raw transcriptomic Fastq files of 48 samples among treated and control were cleaned with Trimmomatic. The sequences were uploaded to the R package, and differentially expressed genes were identified using the DESeq2 algorithm [58]. Sequences were aligned against an in-house oil palm genome with TopHat2 software [59]. The “SummarizeOverlaps” function was used to count overlapping sequences aligned against an in-house oil palm reference genome. A *p-value* ≤ 0.1 and a |*Log2FoldChange| ≥* 2 were set in the DESeq2 algorithm as thresholds for significant differential expression analysis. Al-free hydroponic solution samples for each genotype were considered as control. PCA and heat maps were used to group samples and to identify differentially expressed genes. Gene ontology enrichment analyses were performed with REVIGO [60], using 0.7 medium sizings, *Oryza sativa* Japonica group as the closest relative organism, and SimRel as semantic similarity measurement.

The *igraph* R package was used to construct the global and cultivar-specific co-expression networks. Each network was built based on the correlation of all gene pairs distancing among normalized read counts, and only differentially expressed genes were used. The edge among genes was considered when the correlation was ≥ 0.8. The “edge betweenness” function identified communities or modules of genes correlated among them [61]. HUB genes were determined according to a HUB score ≥ 0.1 using the algorithm developed by Kleinberg [62]. Additional metrics of the networks were estimated by density, diameter [63], diameter (weighted), centralization degree, centralization closeness, centralization betweenness [64, 65], and average path length [66].

### Validation of DEGs by quantitative real-time PCR

The RNA-Seq transcriptome analysis was confirmed with 12 selected genes by RT-qPCR. The genes were selected based on the contrasting expression among cultivars and relevance to the gene co-expression network (genes and normalizer sequences are listed in Additional file 2 supplementary Table 9). The program Primer3web V4.1.0 (https://primer3.ut.ee/) [67] was used to design primer sequences following the minimum information for publication of quantitative real-time PCR experiments (MIQE) guidelines [68]. The cDNA synthesis was achieved using SuperScript™ IV Reverse Transcriptase (Invitrogen™, Massachusetts, United States) following manufacturer recommendations starting from 1 µg of RNA treated with DNase I (Invitrogen™, Massachusetts, United States) as a template. Genes with efficiencies greater than 85% and one defined melting peak were validated. The qPCR reaction was set up in a 10 µL using Fast Evagreen® qPCR Master Mix (Biotium, Inc. Fremont, CA, United States) following manufacture recommendations. The real-time ROCHE LightCycler® 480 System (Roche Diagnostics International AG, Rotkreuz, Switzerland) was set as follows: initial denaturation at 95 °C for 3 min, 40 cycles of denaturation at 95 °C for 10 s, annealing at 57 °C for 30 s and extension at 72 °C for 30 s. Relative expression for each gene was calculated using the delta-delta of Ct method (∆∆Ct), and the NADH gene was used as a normalizer. The association between RNA-Seq and RT-qPCR results was established by a correlation coefficient (r).

### Statistical analyses

The number of tips, stained area, and total length of roots were analyzed using R statistical software and subjected to a two-way analysis of variance (ANOVA). Tukey’s multiple comparison test was used to compare whether means among treatments and differences at *p-value* < 0.05 were considered significant. A non-parametric Kruskal-Wallis Test (*p-value* < 0.05) was performed to compare whether means of NADH expression Ct´s was stable among treatments, experimental conditions, and across genotypes.

## Electronic supplementary material

Below is the link to the electronic supplementary material.

Supplementary Material 1

Supplementary Material 2

## Data Availability

The datasets generated and analyzed during the current study are available in SRA under Bioproject PRJNA892485.
