# Supplementary material for: Molecular network of the oil palm root response to aluminum stress
Source: BMC Plant Biol. 2023 Jun 30;23:346. doi: 10.1186/s12870-023-04354-0 (PMC10311834; doi:10.1186/s12870-023-04354-0)

**Supplementary Materials**

**Molecular network of the oil palm root response to aluminum stress**

**Fernan Santiago Mejía-Alvarado^1^, David Botero-Rozo^1^, Leonardo Araque^1^, Cristihian Bayona^1^, Mariana Herrera-Corzo^1^, Carmenza Montoya^1^, Iván Ayala-Díaz^1^ and Hernán Mauricio Romero^1,2*^.**

**^1^** Colombian Oil Palm Research Center - Cenipalma, Oil Palm Biology, and Breeding Research Program, Bogotá 11121, Colombia.

**^2^** Department of Biology, Universidad Nacional de Colombia, Bogotá 11132, Colombia.

***** Correspondence: hmromeroa@unal.edu.co

**Supplementary Table 2.** Summary metrics of general and specific cultivar gene co-expression networks.

| Network | No. Nodes | Modularity | Edge Density | Diameter | Diameter (weighted) | Cen. (*) Degree | Cen. (*) Closeness | Cen. (*) Betweenness | Average path length |
| --- | --- | --- | --- | --- | --- | --- | --- | --- | --- |
| General | 93 | 0.84 | 19 | 17 | 13.95 | 24 | 12 | 0.18 | 7.38 |
| IRHO 7001 | 104 | 0.81 | 19 | 22 | 17.70 | 29 | 94 | 0.52 | 10.10 |
| CTR 3-0-12 | 31 | 0.65 | 65 | 14 | 11.60 | 102 | 117 | 0.40 | 6.07 |
| CR10-0-2 | 16 | 0.55 | 117 | 6 | 5.24 | 150 | 131 | 0.38 | 3.12 |
| CD 19-12 | 18 | 0.53 | 111 | 8 | 6.78 | 183 | 223 | 0.49 | 3.77 |

Modularity: value of the community structure, modules which are densely connected themselves but sparsely connected to other modules; Density: The proportion of present edges from all possible edges in the network; Diameter: The longest geodesic distance (length of the shortest path between two nodes) in the network; Diameter (weighted): Using a positive weight vector for calculating weighted distances; Centralization Degree: The graph level centrality index (according to the degrees of vertices) normalized by the maximum theoretical graph level centralization score for a graph with the given number of vertices; Centralization Closeness: Inverse of the node’s average geodesic distance to others in the network; Centralization Betweenness: The graph level centrality index based on the number of geodesics that pass through each node or edge; Average path length: The mean of the shortest distance between each pair of nodes in the network. *Cen. = Centralization.

**Supplementary Table 3**. Metrics of the general co-expression network and HUB genes. ^1^Com=Community; ^2^Deg=Degree; ^3^HB=Hub Score; ^4^Bet= Betweenness; ^5^Clos=Closeness.

| Gene | ^1^Com | ^2^Deg | ^3^HS | ^4^Bet | ^5^Clos | Description |
| --- | --- | --- | --- | --- | --- | --- |
| EG01_06G005040 | 4 | 4 | 1 | 572 | 0.000258 | Bowman-Birk type trypsin inhibitor-like |
| EG01_05G004160 | 6 | 4 | 0.86705 | 387 | 0.000256 | phytoene synthase 2 2C chloroplastic-like |
| EG01_14G002750 | 2 | 3 | 0.82227 | 764 | 0.000260 | Putative protein phosphatase 2C 75 isoform X1 |
| EG01_08G007390 | 9 | 4 | 0.77126 | 852 | 0.000262 | pleiotropic drug resistance protein 3-like |
| EG01_U01G039070 | 9 | 4 | 0.74742 | 849 | 0.000263 | Conserved hypothetical protein |
| EG01_11G001260 | 2 | 3 | 0.56213 | 725 | 0.000261 | Putative protein |
| EG01_09G003240 | 11 | 3 | 0.54336 | 540 | 0.000260 | Conserved hypothetical protein |
| EG01_06G009410 | 2 | 3 | 0.50857 | 251 | 0.000257 | inositol-3-phosphate synthase |
| EG01_03G006390 | 4 | 2 | 0.48578 | 104 | 0.000255 | glucan endo-1 2 C3-beta-glucosidase-like |
| EG01_15G001950 | 2 | 2 | 0.44625 | 720 | 0.000262 | vacuolar iron transporter 1.1-like |
| EG01_01G017320 | 6 | 2 | 0.43402 | 153 | 0.000253 | late embryogenesis abundant protein D-34-like |
| EG01_U01G019510 | 6 | 2 | 0.41237 | 104 | 0.000253 | ferritin-4 2C chloroplastic-like |
| EG01_01G009250 | 2 | 2 | 0.40043 | 713 | 0.000262 | Protein ABA deficient 4 2C chloroplastic |
| EG01_01G015730 | 4 | 1 | 0.39710 | 0 | 0.000255 | vacuolar iron transporter 1.1-like |
| EG01_07G009500 | 7 | 2 | 0.38677 | 368 | 0.000260 | hippocampus abundant transcript-like protein 1 |
| EG01_08G007400 | 9 | 2 | 0.37414 | 153 | 0.000259 | pleiotropic drug resistance protein 3-like |
| EG01_07G006070 | 6 | 1 | 0.33708 | 0 | 0.000253 | Coiled-coil domain-containing protein 18 2C |
| EG01_07G007330 | 11 | 3 | 0.33607 | 251 | 0.000257 | chlorophyllide a oxygenase 2C chloroplastic-like isoform X3 |
| EG01_U02G010780 | 9 | 1 | 0.30626 | 0 | 0.000258 | transcription factor bHLH100-like |
| EG01_12G004180 | 9 | 1 | 0.29058 | 0 | 0.000259 | ferritin-4 2C chloroplastic |
| EG01_U02G024250 | 11 | 2 | 0.26101 | 245 | 0.000257 | metal transporter Nramp2-like |
| EG01_U03G012710 | 6 | 3 | 0.24933 | 105 | 0.000250 | phosphoenolpyruvate carboxykinase [ATP] |
| EG01_04G014350 | 2 | 2 | 0.24706 | 104 | 0.000254 | major latex allergen Hev b 5-like |
| EG01_03G005920 | 2 | 2 | 0.23882 | 53 | 0.000254 | glutaredoxin-C1-like |
| EG01_04G011590 | 4 | 2 | 0.22334 | 53 | 0.000252 | protein NRT1 PTR FAMILY 6.4 |
| EG01_14G003610 | 2 | 1 | 0.22322 | 0 | 0.000258 | photosystem I reaction center subunit IV B 2C chloroplastic-like |
| EG01_U01G010130 | 9 | 3 | 0.21493 | 105 | 0.000256 | oligopeptide transporter 3-like |
| EG01_U02G015940 | 7 | 2 | 0.20275 | 329 | 0.000257 | metal transporter Nramp5-like |
| EG01_13G006740 | 6 | 2 | 0.19365 | 53 | 0.000250 | rhodanese-like domain-containing protein 15 2C chloroplastic |
| EG01_U03G020670 | 11 | 2 | 0.16326 | 104 | 0.000254 | NAC domain-containing protein 68-like |
| EG01_U01G034620 | 11 | 2 | 0.15781 | 53 | 0.000254 | Conserved hypothetical protein |
| EG01_09G003350 | 7 | 3 | 0.13474 | 293 | 0.000254 | Doubtful hypothetical protein |
| EG01_U02G023550 | 11 | 2 | 0.12801 | 200 | 0.000254 | NAC domain-containing protein 68-like |
| EG01_16G001480 | 2 | 2 | 0.11358 | 53 | 0.000251 | peroxidase 15-like |
| EG01_05G003640 | 6 | 1 | 0.09693 | 0 | 0.000247 | protein FEZ-like |
| EG01_15G000600 | 6 | 1 | 0.09693 | 0 | 0.000247 | magnesium transporter MRS2-I-like |
| EG01_U03G016900 | 2 | 1 | 0.09285 | 0 | 0.000251 | Putative LRR receptor-like serine |
| EG01_02G003010 | 4 | 1 | 0.08869 | 0 | 0.000249 | ninja-family protein AFP3-like |
| EG01_10G001860 | 7 | 3 | 0.08418 | 203 | 0.000251 | GDP-mannose 3 2C5-epimerase 2-like |
| EG01_04G020660 | 9 | 1 | 0.08356 | 0 | 0.000253 | two-component response regulator-like APRR3 |
| EG01_U01G006470 | 9 | 1 | 0.08356 | 0 | 0.000253 | Putative amino-acid acetyltransferase NAGS 2C chloroplastic isoform X1 |
| EG01_U03G007270 | 6 | 1 | 0.07528 | 0 | 0.000246 | mannose glucose-specific lectin-like isoform X1 crll |
| EG01_07G005820 | 11 | 2 | 0.07506 | 53 | 0.000251 | Sucrose-phosphate synthase |
| EG01_07G009960 | 11 | 1 | 0.06135 | 0 | 0.000251 | hippocampus abundant transcript-like protein 1 |
| EG01_10G004400 | 11 | 2 | 0.06135 | 153 | 0.000251 | fe(2+) transport protein 1-like |
| EG01_U01G008360 | 7 | 1 | 0.05238 | 0 | 0.000251 | ethylene-responsive transcription factor ERF113-like |
| EG01_02G003410 | 7 | 3 | 0.04836 | 105 | 0.000248 | Auxin efflux carrier component 5 |
| EG01_U03G019210 | 2 | 1 | 0.04510 | 0 | 0.000248 | zinc finger protein STOP1 homolog |
| EG01_02G013870 | 7 | 1 | 0.03343 | 0 | 0.000248 | zinc finger protein 622-like |
| EG01_08G007380 | 11 | 2 | 0.02981 | 104 | 0.000248 | chalcone synthase 3-like |
| EG01_12G002190 | 11 | 1 | 0.02981 | 0 | 0.000247 | P-hydroxybenzoic acid efflux pump subunit aaeB |
| EG01_06G007260 | 7 | 1 | 0.01880 | 0 | 0.000245 | metal tolerance protein 7-like |
| EG01_U03G019530 | 7 | 1 | 0.01880 | 0 | 0.000245 | E3 SUMO-protein ligase RanBP2-like |
| EG01_10G004410 | 11 | 2 | 0.01370 | 53 | 0.000245 | fe(2+) transport protein 1-like |
| EG01_15G006910 | 11 | 1 | 0.00544 | 0 | 0.000242 | zinc finger CCCH domain-containing protein 45-like isoform X1 |
| EG01_08G018760 | 12 | 1 | 0.00000 | 0 | 0.000118 | early light-induced protein 1 2C chloroplastic-like isoform X1 |
| EG01_10G002000 | 12 | 1 | 0.00000 | 0 | 0.000118 | early light-induced protein 1 2C chloroplastic-like |
| EG01_10G002380 | 13 | 1 | 0.00000 | 0 | 0.000118 | cytochrome P450 78A5-like |
| EG01_15G007370 | 14 | 1 | 0.00000 | 0 | 0.000118 | cysteine-rich and transmembrane domain-containing protein A |
| EG01_U01G009050 | 15 | 1 | 0.00000 | 0 | 0.000118 | Os03g0645966 protein (Fragment) |
| EG01_U01G009060 | 15 | 1 | 0.00000 | 0 | 0.000118 | 9-cis-epoxycarotenoid dioxygenase 2C chloroplastic-like |
| EG01_U02G015600 | 13 | 1 | 0.00000 | 0 | 0.000118 | LRR receptor-like serine threonine-protein kinase GSO1 F775_15117 |
| EG01_U02G026080 | 14 | 1 | 0.00000 | 0 | 0.000118 | cysteine-rich and transmembrane domain-containing protein A |
| EG01_01G001210 | 1 | 2 | 0.00000 | 12 | 0.000128 | type I inositol 1 2C4 2C5-trisphosphate 5-phosphatase 1 |
| EG01_01G002300 | 1 | 1 | 0.00000 | 0 | 0.000128 | diacylglycerol O-acyltransferase 1-like isoform X2 |
| EG01_01G014480 | 3 | 2 | 0.00000 | 5 | 0.000125 | Doubtful hypothetical protein |
| EG01_01G016740 | 5 | 2 | 0.00000 | 4 | 0.000123 | calcium-transporting ATPase 8 2C plasma membrane-type-like isoform X1 |
| EG01_01G018550 | 3 | 1 | 0.00000 | 0 | 0.000125 | disease resistance RPP8-like protein 3 |
| EG01_01G018560 | 3 | 2 | 0.00000 | 5 | 0.000125 | Putative disease resistance RPP8-like protein 2 |
| EG01_02G005450 | 5 | 1 | 0.00000 | 0 | 0.000123 | Gamma-glutamyl peptidase 5 |
| EG01_03G003190 | 1 | 1 | 0.00000 | 0 | 0.000128 | peroxidase P7-like |
| EG01_04G010230 | 5 | 1 | 0.00000 | 0 | 0.000123 | ethylene-responsive transcription factor ERF027-like |
| EG01_04G016430 | 8 | 1 | 0.00000 | 0 | 0.000121 | Conserved hypothetical protein |
| EG01_05G000670 | 3 | 1 | 0.00000 | 0 | 0.000125 | chlorophyll a-b binding protein 4 2C chloroplastic |
| EG01_06G008660 | 10 | 1 | 0.00000 | 0 | 0.000121 | calmodulin-like |
| EG01_08G017690 | 10 | 1 | 0.00000 | 0 | 0.000121 | protein PHLOEM PROTEIN 2-LIKE A1-like |
| EG01_08G018520 | 1 | 1 | 0.00000 | 0 | 0.000128 | tryptophan synthase alpha chain-like |
| EG01_09G009480 | 8 | 3 | 0.00000 | 3 | 0.000121 | nuclear transcription factor Y subunit B-3-like |
| EG01_10G006680 | 5 | 4 | 0.00000 | 9 | 0.000124 | gibberellin 2-beta-dioxygenase 8 isoform X2 |
| EG01_10G008040 | 1 | 1 | 0.00000 | 0 | 0.000128 | Jasmonic acid-amido synthetase JAR1 |
| EG01_11G004380 | 10 | 2 | 0.00000 | 2 | 0.000121 | NAC domain-containing protein 72-like isoform X1 |
| EG01_12G010370 | 1 | 4 | 0.00000 | 22 | 0.000128 | protein LURP-one-related 11-like |
| EG01_12G012660 | 5 | 1 | 0.00000 | 0 | 0.000123 | peroxidase 5-like |
| EG01_15G001270 | 10 | 2 | 0.00000 | 2 | 0.000121 | basic blue protein-like |
| EG01_15G007360 | 3 | 2 | 0.00000 | 8 | 0.000125 | cysteine-rich and transmembrane domain-containing protein A |
| EG01_U01G018670 | 8 | 1 | 0.00000 | 0 | 0.000121 | ankyrin repeat-containing protein At3g12360-like |
| EG01_U01G025080 | 8 | 1 | 0.00000 | 0 | 0.000121 | Xyloglucan endotransglucosylase hydrolase protein A |
| EG01_U02G020710 | 3 | 1 | 0.00000 | 0 | 0.000125 | Conserved hypothetical protein |
| EG01_U03G001880 | 3 | 3 | 0.00000 | 11 | 0.000125 | Conserved hypothetical protein |
| EG01_U03G010530 | 1 | 2 | 0.00000 | 7 | 0.000128 | E3 ubiquitin-protein ligase PUB23-like |
| EG01_U03G013910 | 5 | 1 | 0.00000 | 0 | 0.000123 | Calcium-binding protein CML38 |
| EG01_U03G018460 | 1 | 2 | 0.00000 | 12 | 0.000128 | Doubtful hypothetical protein |
| EG01_U03G018720 | 1 | 2 | 0.00000 | 7 | 0.000128 | trans-resveratrol di-O-methyltransferase-like |

**Supplementary Table 4**. Metrics of the IRHO 7001 co-expression network and HUB genes. ^1^Com=Community; ^2^Deg=Degree; ^3^HB=Hub Score; ^4^Bet= Betweenness; ^5^Clos=Closeness.

| Gene | ^1^Com | ^2^Deg | ^3^HS | ^4^Bet | ^5^Clos | Description |
| --- | --- | --- | --- | --- | --- | --- |
| EG01_08G007400 | 11 | 5 | 1 | 1385 | 0.001028 | pleiotropic drug resistance protein 3-like |
| EG01_U01G006470 | 4 | 5 | 0.83586 | 781 | 0.000944 | Putative amino-acid acetyltransferase NAGS1 2C chloroplastic isoform X1 |
| EG01_16G001480 | 10 | 4 | 0.72482 | 2238 | 0.001110 | peroxidase 15-like |
| EG01_07G007330 | 4 | 4 | 0.50183 | 303 | 0.000866 | chlorophyllide a oxygenase 2C chloroplastic-like isoform X3 |
| EG01_10G001860 | 10 | 4 | 0.49486 | 866 | 0.001015 | GDP-mannose 3 2C5-epimerase 2-like |
| EG01_16G005800 | 11 | 2 | 0.39302 | 202 | 0.000934 | cytochrome P450 78A5-like |
| EG01_08G007390 | 3 | 3 | 0.38834 | 2246 | 0.001172 | pleiotropic drug resistance protein 3-like |
| EG01_13G006990 | 11 | 2 | 0.38489 | 102 | 0.000932 | Conserved hypothetical protein |
| EG01_11G004380 | 4 | 2 | 0.35742 | 102 | 0.000863 | NAC domain-containing protein 72-like isoform X1 |
| EG01_10G008040 | 11 | 1 | 0.33647 | 0 | 0.000930 | Jasmonic acid-amido synthetase JAR1 |
| EG01_01G018560 | 4 | 1 | 0.31246 | 0 | 0.000861 | Putative disease resistance RPP8-like protein 2 |
| EG01_U01G025080 | 4 | 1 | 0.31246 | 0 | 0.000861 | Xyloglucan endotransglucosylase hydrolase protein A |
| EG01_U02G024250 | 10 | 1 | 0.27095 | 0 | 0.000997 | metal transporter Nramp2-like |
| EG01_U01G010620 | 10 | 3 | 0.24200 | 496 | 0.000929 | UPF0481 protein At3g47200-like |
| EG01_U01G009060 | 10 | 2 | 0.19047 | 102 | 0.000922 | 9-cis-epoxycarotenoid dioxygenase 2C chloroplastic-like |
| EG01_01G015730 | 4 | 1 | 0.16885 | 0 | 0.000796 | vacuolar iron transporter 1.1-like |
| EG01_08G006650 | 4 | 1 | 0.16885 | 0 | 0.000796 | dynein light chain LC6 2C flagellar outer arm-like |
| EG01_U03G026740 | 4 | 1 | 0.16885 | 0 | 0.000796 | alpha-humulene synthase-like |
| EG01_08G017690 | 11 | 2 | 0.16806 | 102 | 0.000854 | protein PHLOEM PROTEIN 2-LIKE A1-like |
| EG01_15G006910 | 10 | 1 | 0.16651 | 0 | 0.000920 | zinc finger CCCH domain-containing protein 45-like isoform X1 |
| EG01_14G001570 | 3 | 2 | 0.16454 | 2232 | 0.001233 | BURP domain-containing protein 6-like |
| EG01_02G011360 | 3 | 2 | 0.14947 | 102 | 0.001049 | 11 kDa late embryogenesis abundant protein-like |
| EG01_U01G039070 | 11 | 1 | 0.14388 | 0 | 0.000851 | Conserved hypothetical protein |
| EG01_15G001270 | 10 | 3 | 0.12087 | 203 | 0.000851 | basic blue protein-like |
| EG01_01G014320 | 4 | 1 | 0.12026 | 0 | 0.000793 | Conserved hypothetical protein |
| EG01_07G002770 | 10 | 2 | 0.10348 | 102 | 0.000850 | LOW QUALITY PROTEIN: Putative disease resistance protein At1g58602 |
| EG01_08G007380 | 3 | 3 | 0.10068 | 2720 | 0.001297 | chalcone synthase 3-like |
| EG01_10G002000 | 10 | 1 | 0.07120 | 0 | 0.000842 | early light-induced protein 1 2C chloroplastic-like |
| EG01_04G021290 | 9 | 4 | 0.05817 | 2702 | 0.001339 | Hydrolase 2C alpha beta fold family protein 2C Putative 2C |
| EG01_U01G010540 | 11 | 1 | 0.05655 | 0 | 0.000786 | polygalacturonate 4-alpha-galacturonosyltransferase-like |
| EG01_10G004400 | 3 | 1 | 0.05587 | 0 | 0.000948 | fe(2+) transport protein 1-like |
| EG01_01G013640 | 3 | 3 | 0.04662 | 587 | 0.001161 | MLO-like protein 2 2C partial |
| EG01_04G003290 | 10 | 1 | 0.04067 | 0 | 0.000783 | subtilisin-like protease |
| EG01_U02G012860 | 10 | 1 | 0.04067 | 0 | 0.000783 | alpha-humulene synthase-like |
| EG01_10G009580 | 10 | 1 | 0.03482 | 0 | 0.000782 | ABC transporter G family member 25-like |
| EG01_15G000600 | 9 | 2 | 0.02558 | 2596 | 0.001368 | magnesium transporter MRS2-I-like |
| EG01_02G003410 | 9 | 2 | 0.02487 | 102 | 0.001181 | Auxin efflux carrier component 5 |
| EG01_U02G015940 | 9 | 1 | 0.02175 | 0 | 0.001178 | metal transporter Nramp5-like |
| EG01_U01G010130 | 3 | 2 | 0.02044 | 396 | 0.001047 | oligopeptide transporter 3-like |
| EG01_07G009960 | 3 | 1 | 0.01743 | 0 | 0.001038 | hippocampus abundant transcript-like protein 1 |
| EG01_U02G002270 | 9 | 2 | 0.01026 | 2610 | 0.001395 | TPD1 protein homolog 1 |
| EG01_U03G020670 | 9 | 1 | 0.00837 | 0 | 0.001054 | NAC domain-containing protein 68-like |
| EG01_07G006070 | 3 | 2 | 0.00806 | 300 | 0.000951 | Coiled-coil domain-containing protein 18 2C Putative isoform 2 |
| EG01_04G011590 | 9 | 2 | 0.00491 | 2622 | 0.001418 | protein NRT1 PTR FAMILY 6.4 VIT_12s0059g01240 |
| EG01_12G004180 | 3 | 2 | 0.00352 | 202 | 0.000870 | ferritin-4 2C chloroplastic |
| EG01_03G006390 | 9 | 3 | 0.00287 | 3016 | 0.001439 | glucan endo-1 2C3-beta-glucosidase-like |
| EG01_U02G020840 | 6 | 4 | 0.00235 | 3188 | 0.001423 | short-chain dehydrogenase TIC 32 2C chloroplastic-like isoform X1 |
| EG01_04G014350 | 3 | 2 | 0.00136 | 102 | 0.000801 | major latex allergen Hev b 5-like |
| EG01_U02G023550 | 6 | 3 | 0.00132 | 2351 | 0.001342 | NAC domain-containing protein 68-like |
| EG01_04G020660 | 5 | 2 | 0.00128 | 672 | 0.001277 | two-component response regulator-like APRR3 |
| EG01_U01G009050 | 8 | 3 | 0.00116 | 854 | 0.001271 | Os03g0645966 protein (Fragment) |
| EG01_14G002750 | 6 | 2 | 0.00093 | 490 | 0.001258 | Putative protein phosphatase 2C 75 isoform X1 |
| EG01_06G008660 | 7 | 3 | 0.00079 | 587 | 0.001198 | calmodulin-like |
| EG01_13G006740 | 1 | 3 | 0.00078 | 1902 | 0.001245 | rhodanese-like domain-containing protein 15 2C chloroplastic |
| EG01_U02G015050 | 8 | 3 | 0.00067 | 682 | 0.001143 | heavy metal-associated isoprenylated plant protein 26-like |
| EG01_06G005040 | 5 | 2 | 0.00055 | 582 | 0.001146 | Bowman-Birk type trypsin inhibitor-like |
| EG01_04G017230 | 7 | 5 | 0.00054 | 402 | 0.001076 | Retrovirus-related Pol polyprotein from transposon 17.6 |
| EG01_08G018520 | 3 | 1 | 0.00051 | 0 | 0.000740 | tryptophan synthase alpha chain-like |
| EG01_15G000510 | 1 | 3 | 0.00044 | 866 | 0.001127 | serine carboxypeptidase-like 40 isoform X3 |
| EG01_08G018760 | 8 | 1 | 0.00043 | 0 | 0.001125 | early light-induced protein 1 2C chloroplastic-like isoform X1 |
| EG01_10G011540 | 6 | 2 | 0.00041 | 396 | 0.001125 | pistil-specific extensin-like protein |
| EG01_05G003640 | 8 | 4 | 0.00037 | 401 | 0.001032 | protein FEZ-like |
| EG01_14G003610 | 5 | 4 | 0.00035 | 498 | 0.001036 | photosystem I reaction center subunit IV B 2C chloroplastic-like |
| EG01_09G003240 | 1 | 2 | 0.00032 | 1012 | 0.001133 | Conserved hypothetical protein |
| EG01_06G007260 | 1 | 4 | 0.00028 | 401 | 0.001019 | metal tolerance protein 7-like |
| EG01_05G004160 | 7 | 1 | 0.00027 | 0 | 0.001067 | phytoene synthase 2 2C chloroplastic-like |
| EG01_U02G010780 | 1 | 4 | 0.00027 | 303 | 0.001017 | transcription factor bHLH100-like |
| EG01_U03G019210 | 8 | 2 | 0.00026 | 102 | 0.001026 | zinc finger protein STOP1 homolog |
| EG01_01G018550 | 7 | 1 | 0.00020 | 0 | 0.000970 | disease resistance RPP8-like protein 3 |
| EG01_02G004590 | 7 | 1 | 0.00020 | 0 | 0.000970 | polyphenol oxidase 2C chloroplastic-like |
| EG01_09G009480 | 7 | 1 | 0.00020 | 0 | 0.000970 | nuclear transcription factor Y subunit B-3-like |
| EG01_U03G001880 | 7 | 1 | 0.00020 | 0 | 0.000970 | Conserved hypothetical protein |
| EG01_11G001260 | 2 | 3 | 0.00018 | 951 | 0.001036 | Putative protein |
| EG01_07G002710 | 6 | 2 | 0.00017 | 300 | 0.001015 | ferric reduction oxidase 8 2C mitochondrial |
| EG01_07G009500 | 8 | 2 | 0.00016 | 102 | 0.000935 | hippocampus abundant transcript-like protein 1 |
| EG01_02G003010 | 8 | 1 | 0.00014 | 0 | 0.000934 | ninja-family protein AFP3-like |
| EG01_U01G034620 | 8 | 1 | 0.00014 | 0 | 0.000934 | Conserved hypothetical protein |
| EG01_01G014480 | 5 | 2 | 0.00013 | 102 | 0.000939 | Doubtful hypothetical protein |
| EG01_07G005820 | 5 | 2 | 0.00013 | 102 | 0.000939 | Sucrose-phosphate synthase |
| EG01_U01G001510 | 5 | 1 | 0.00012 | 0 | 0.000937 | cytochrome P450 78A5-like |
| EG01_01G011560 | 1 | 2 | 0.00011 | 102 | 0.000925 | inositol-3-phosphate synthase-like |
| EG01_08G009080 | 8 | 1 | 0.00010 | 0 | 0.000929 | dnaJ homolog subfamily B member 3-like |
| EG01_02G013870 | 1 | 1 | 0.00009 | 0 | 0.000923 | zinc finger protein 622-like |
| EG01_U03G019530 | 1 | 1 | 0.00009 | 0 | 0.000923 | E3 SUMO-protein ligase RanBP2-like |
| EG01_01G009250 | 1 | 1 | 0.00009 | 0 | 0.000922 | Protein ABA DEFICIENT 4 2C chloroplastic |
| EG01_05G005980 | 1 | 1 | 0.00009 | 0 | 0.000922 | glycine-rich cell wall structural protein 2-like |
| EG01_U01G019510 | 1 | 1 | 0.00009 | 0 | 0.000922 | ferritin-4 2C chloroplastic-like |
| EG01_07G009190 | 6 | 3 | 0.00008 | 203 | 0.000923 | disease resistance protein RPP13-like |
| EG01_10G005440 | 2 | 3 | 0.00008 | 203 | 0.000941 | Conserved hypothetical protein |
| EG01_03G005920 | 2 | 2 | 0.00007 | 582 | 0.000948 | glutaredoxin-C1-like |
| EG01_U01G008360 | 8 | 1 | 0.00005 | 0 | 0.000854 | ethylene-responsive transcription factor ERF113-like 2Cpartial |
| EG01_06G009410 | 5 | 1 | 0.00005 | 0 | 0.000857 | inositol-3-phosphate synthase |
| EG01_15G001950 | 5 | 1 | 0.00005 | 0 | 0.000857 | vacuolar iron transporter 1.1-like |
| EG01_U02G026940 | 1 | 1 | 0.00004 | 0 | 0.000845 | protein MKS1-like |
| EG01_10G004410 | 2 | 3 | 0.00004 | 494 | 0.000872 | fe(2+) transport protein 1-like |
| EG01_U03G007270 | 2 | 1 | 0.00003 | 0 | 0.000858 | mannose glucose-specific lectin-like isoform X1 crll |
| EG01_U03G016900 | 2 | 1 | 0.00003 | 0 | 0.000858 | Putative LRR receptor-like serine |
| EG01_01G017320 | 6 | 1 | 0.00003 | 0 | 0.000844 | late embryogenesis abundant protein D-34-like |
| EG01_03G003190 | 6 | 1 | 0.00003 | 0 | 0.000844 | peroxidase P7-like |
| EG01_U03G012710 | 2 | 3 | 0.00002 | 302 | 0.000805 | phosphoenolpyruvate carboxykinase [ATP] |
| EG01_01G013440 | 2 | 1 | 0.00001 | 0 | 0.000801 | E3 ubiquitin-protein ligase RNF14-like |
| EG01_U01G010940 | 2 | 2 | 0.00001 | 102 | 0.000745 | Bowman-Birk type trypsin inhibitor-like |
| EG01_06G001010 | 2 | 1 | 0.00001 | 0 | 0.000743 | protein GIGANTEA-like |
| EG01_04G016430 | 2 | 1 | 0.00000 | 0 | 0.000692 | Conserved hypothetical protein |

**Supplementary Table 5**. Metrics of the CTR 3-0-12 co-expression network and HUB genes. ^1^Com=Community; ^2^Deg=Degree; ^3^HB=Hub Score; ^4^Bet= Betweenness; ^5^Clos=Closeness.

| Gene | ^1^Com | ^2^Deg | ^3^HS | ^4^Bet | ^5^Clos | Description |
| --- | --- | --- | --- | --- | --- | --- |
| EG01_U03G009060 | 3 | 5 | 1 | 156 | 0.005917 | dehydration-responsive element-binding protein 1F-like |
| EG01_10G006680 | 5 | 4 | 0.73837 | 245 | 0.007194 | gibberellin 2-beta-dioxygenase 8 isoform X2 |
| EG01_U02G020710 | 3 | 3 | 0.66631 | 183 | 0.006579 | Conserved hypothetical protein |
| EG01_01G002300 | 2 | 3 | 0.46849 | 57 | 0.005155 | diacylglycerol O-acyltransferase 1-like isoform X2 |
| EG01_U03G013910 | 5 | 3 | 0.36751 | 237 | 0.007463 | Calcium-binding protein CML38 |
| EG01_02G005450 | 3 | 1 | 0.32432 | 0 | 0.005051 | Gamma-glutamyl peptidase 5 |
| EG01_12G012660 | 3 | 1 | 0.32432 | 0 | 0.005051 | peroxidase 5-like |
| EG01_U03G018720 | 3 | 1 | 0.32432 | 0 | 0.005051 | trans-resveratrol di-O-methyltransferase-like |
| EG01_12G013990 | 3 | 1 | 0.31612 | 0 | 0.005525 | protein LURP-one-related 10-like |
| EG01_10G003820 | 5 | 2 | 0.28301 | 29 | 0.006024 | disease resistance protein RGA2-like 2C partial |
| EG01_15G007360 | 5 | 1 | 0.23947 | 0 | 0.005952 | cysteine-rich and transmembrane domain-containing protein A |
| EG01_U02G018650 | 2 | 1 | 0.22227 | 0 | 0.004484 | LOW QUALITY PROTEIN: Putative disease resistance protein At1g12280 |
| EG01_U02G020500 | 2 | 1 | 0.22227 | 0 | 0.004484 | polyol transporter 5-like |
| EG01_U03G018460 | 5 | 2 | 0.22045 | 225 | 0.007519 | Doubtful hypothetical protein |
| EG01_04G024930 | 5 | 1 | 0.17436 | 0 | 0.006135 | cinnamoyl-CoA reductase 2-like |
| EG01_07G002200 | 5 | 1 | 0.13427 | 0 | 0.005128 | momilactone A synthase-like isoform X1 |
| EG01_12G002190 | 4 | 2 | 0.09714 | 224 | 0.007463 | P-hydroxybenzoic acid efflux pump subunit aaeB |
| EG01_04G010230 | 4 | 3 | 0.07907 | 233 | 0.007299 | ethylene-responsive transcription factor ERF027-like |
| EG01_13G009300 | 4 | 3 | 0.04388 | 227 | 0.006944 | Putative LRR receptor-like serine |
| EG01_12G010370 | 4 | 2 | 0.03162 | 176 | 0.006369 | protein LURP-one-related 11-like |
| EG01_04G021820 | 4 | 1 | 0.02564 | 0 | 0.006024 | Stress-response A B barrel domain-containing protein At5g22580 |
| EG01_04G018840 | 4 | 2 | 0.02460 | 29 | 0.005848 | phospholipase A1-Ialpha2 2C chloroplastic |
| EG01_15G007370 | 1 | 4 | 0.02276 | 172 | 0.005814 | cysteine-rich and transmembrane domain-containing protein A |
| EG01_01G016740 | 1 | 3 | 0.01697 | 108 | 0.005181 | calcium-transporting ATPase 8 2C plasma membrane-type-like isoform X1 |
| EG01_01G001210 | 1 | 1 | 0.01080 | 0 | 0.004975 | type I inositol 1 2C4 2C5-trisphosphate 5-phosphatase 1 |
| EG01_06G006010 | 1 | 1 | 0.01080 | 0 | 0.004975 | S-type anion channel SLAH4-like |
| EG01_U03G010530 | 4 | 1 | 0.00798 | 0 | 0.005000 | E3 ubiquitin-protein ligase PUB23-like |
| EG01_10G000710 | 1 | 2 | 0.00651 | 29 | 0.004546 | protein SULFUR DEFICIENCY-INDUCED 1-like |
| EG01_U02G026080 | 1 | 2 | 0.00651 | 29 | 0.004546 | cysteine-rich and transmembrane domain-containing protein A |
| EG01_11G001760 | 1 | 1 | 0.00309 | 0 | 0.004016 | Pyruvate dehydrogenase E1 component subunit alpha |
| EG01_16G003030 | 1 | 1 | 0.00309 | 0 | 0.004016 | heavy metal-associated isoprenylated plant protein 26-like |

**Supplementary Table 6**. Metrics of the CR10-0-2 co-expression network and HUB genes. ^1^Com=Community; ^2^Deg=Degree; ^3^HB=Hub Score; ^4^Bet= Betweenness; ^5^Clos=Closeness.

| Gene | ^1^Com | ^2^Deg | ^3^HS | ^4^Bet | ^5^Clos | Description |
| --- | --- | --- | --- | --- | --- | --- |
| EG01_U02G010780 | 1 | 5 | 1 | 78 | 0.020408 | transcription factor bHLH100-like |
| EG01_U01G010130 | 2 | 2 | 0.55364 | 72 | 0.022222 | oligopeptide transporter 3-like |
| EG01_U01G019510 | 3 | 3 | 0.37059 | 31 | 0.016949 | ferritin-4 2C chloroplastic-like |
| EG01_07G007330 | 1 | 1 | 0.30452 | 0 | 0.016393 | chlorophyllide an oxygenase 2C chloroplastic-like isoform X3 |
| EG01_10G004410 | 2 | 2 | 0.29795 | 16 | 0.016393 | Fe (2+) transport protein 1-like |
| EG01_U03G019690 | 2 | 2 | 0.29795 | 16 | 0.016393 | glycine-rich cell wall structural protein 2-like |
| EG01_09G003240 | 4 | 2 | 0.24379 | 16 | 0.013333 | Conserved hypothetical protein |
| EG01_01G015730 | 1 | 3 | 0.00000 | 79 | 0.022222 | vacuolar iron transporter 1.1-like |
| EG01_02G003410 | 1 | 1 | 0.00000 | 0 | 0.015385 | Auxin efflux carrier component 5 |
| EG01_02G013870 | 1 | 1 | 0.00000 | 0 | 0.015385 | zinc finger protein 622-like |
| EG01_04G014350 | 2 | 1 | 0.00000 | 0 | 0.012987 | major latex allergen Hev b 5-like |
| EG01_06G007260 | 1 | 1 | 0.00000 | 0 | 0.015385 | metal tolerance protein 7-like |
| EG01_07G009500 | 2 | 1 | 0.00000 | 0 | 0.012987 | hippocampus abundant transcript-like protein 1 |
| EG01_07G009960 | 3 | 1 | 0.00000 | 0 | 0.013333 | hippocampus abundant transcript-like protein 1 |
| EG01_08G007390 | 4 | 2 | 0.00000 | 30 | 0.016393 | pleiotropic drug resistance protein 3-like |
| EG01_10G002000 | 3 | 1 | 0.00000 | 0 | 0.013333 | early light-induced protein 1 2C chloroplastic-like |
| EG01_12G004180 | 2 | 4 | 0.00000 | 86 | 0.021277 | ferritin-4 2C chloroplastic |
| EG01_15G001950 | 4 | 1 | 0.00000 | 0 | 0.010989 | vacuolar iron transporter 1.1-like |

**Supplementary Table 7**. Metrics of the CD 19-12 co-expression network and HUB genes. ^1^Com=Community; ^2^Deg=Degree; ^3^HB=Hub Score; ^4^Bet= Betweenness; ^5^Clos=Closeness.

| Gene | ^1^Com | ^2^Deg | ^3^HS | ^4^Bet | ^5^Clos | Description |
| --- | --- | --- | --- | --- | --- | --- |
| EG01_U01G006660 | 3 | 3 | 1 | 47 | 0.016667 | Doubtful hypothetical protein |
| EG01_07G009960 | 4 | 3 | 0.94787 | 50 | 0.016667 | hippocampus abundant transcript-like protein 1 |
| EG01_U02G022510 | 5 | 4 | 0.90490 | 41 | 0.015625 | wall-associated receptor kinase-like 1 2C partial |
| EG01_U01G010130 | 1 | 4 | 0.89107 | 33 | 0.015152 | oligopeptide transporter 3-like |
| EG01_U01G018670 | 4 | 2 | 0.51160 | 12 | 0.014286 | ankyrin repeat-containing protein At3g12360-like |
| EG01_U02G015600 | 5 | 2 | 0.48840 | 12 | 0.013514 | LRR receptor-like serine threonine-protein kinase GSO1 F775_15117 |
| EG01_10G002380 | 5 | 1 | 0.40266 | 0 | 0.013158 | cytochrome P450 78A5-like |
| EG01_U02G006550 | 5 | 1 | 0.40266 | 0 | 0.013158 | protein argonaute MEL1-like isoform X1 |
| EG01_07G005820 | 3 | 1 | 0.39451 | 0 | 0.013889 | Sucrose-phosphate synthase |
| EG01_02G013920 | 1 | 1 | 0.35153 | 0 | 0.012821 | ethylene-responsive transcription factor ERF003-like |
| EG01_09G003350 | 1 | 1 | 0.35153 | 0 | 0.012821 | Doubtful hypothetical protein |
| EG01_12G004180 | 1 | 1 | 0.35153 | 0 | 0.012821 | ferritin-4 2C chloroplastic |
| EG01_U01G020030 | 4 | 1 | 0.20183 | 0 | 0.012195 | Glutathione S-transferase U25 |
| EG01_11G011490 | 5 | 1 | 0.19268 | 0 | 0.011628 | LRR receptor-like serine threonine-protein kinase GSO1 TCM_026241 |
| EG01_03G005620 | 2 | 1 | 0.00000 | 0 | 0.004444 | desiccation-related protein PCC13-62-like isoform X1 |
| EG01_04G014350 | 2 | 1 | 0.00000 | 0 | 0.004444 | major latex allergen Hev b 5-like |

**Supplementary Table 8.** Parents (mother and father) of a commercial cultivar and 3 experimental genotypes from the Cenipalma working collections.

|  | GENOTYPE |  | MOTHER |  | POLLEN SOURCE |  |
| --- | --- | --- | --- | --- | --- | --- |
|  | IRHO 7001 |  | Deli |  | La Mé |  |
|  | CTR 3-0-12 |  | Cameroon |  | Yangambi |  |
|  | CR 10-0-2 |  | Cameroon |  | Yangambi |  |
|  | CD 19-12 |  | Cameroon |  | Yangambi |  |

**Supplementary Table 9**. Primer sequences of genes selected for validation. % Eff. = efficiency percentage.

| Gene ID | Gene | Sequence 5´ → 3´ | Tm (°C) | Size (pb) | % Eff.* |
| --- | --- | --- | --- | --- | --- |
| EG01_U03G009060 | Dehydration-responsive element-binding protein 1F-like | F- AACTCAACTTCCCCGATTCC | 58.4 | 127 | 85 |
|  |  | R- TCCTCGTCCACAAAAACCTC | 58.4 |  |  |
| EG01_11G004380 | NAC domain-containing protein 72-like isoform X1 | F- ATTTCAGCCCAAAAATGTGC | 54.3 | 109 | 104 |
|  |  | R- AGGGAATTGCTTCGAGTTGA | 56.4 |  |  |
| EG01_U03G018720 | Trans-resveratrol di-O-methyltransferase -like | F- AGCCAATCTCCCTCCTTGA | 57.3 | 147 | 105 |
|  |  | R- GCTCTTCGTCGTGGTCGT | 58.4 |  |  |
| EG01_U01G010620 | UPF0481 protein At3g47200-like | F- GGGTGCTTCATCATCCATCT | 58.4 | 154 | 96 |
|  |  | R- GCCGAACTGTGAACAAACCT | 58.4 |  |  |
| EG01_U01G009060 | 9-cis-epoxycarotenoid dioxygenase 2C chloroplastic-like | F- ACTGCTTCTGCTTCCACCTC | 60.5 | 158 | 104 |
|  |  | R- CTTGTTCCGGTTCACCATTC | 58.4 |  |  |
| EG01_08G017690 | Protein phloem protein 2-like A1-like | F- GATCACATGGGGAGATGACC | 60.5 | 98 | 100 |
|  |  | R- CGAGCCAACAAACTTCCAAC | 58.4 |  |  |
| EG01_04G021820 | Stress-response AB barrel domain-containing protein At5g22580 | F- GAATGGGGACGGAATGTG | 56.1 | 120 | 106 |
|  |  | R- GAGGTGGCTGGGATGGTT | 58.4 |  |  |
| EG01_01G015730 | Vacuolar iron transporter 1.1-like | F- AGCCGAGGAGGGCATTAC | 58.4 | 90 | 93 |
|  |  | R- TGATGAACACGTAGGGCAAG | 58.4 |  |  |
| EG01_05G004160 | Phytoene synthase 2 2C chloroplastic-like | F- TGAGCCACAAGAGAATGACAA | 57.4 | 123 | 97 |
|  |  | R- ACCAATGCTGCCTGCTTC | 56.1 |  |  |
| EG01_01G017320 | Late embryogenesis abundant protein D-34-like | F- CGGTTGATGCGTGAGGAG | 58.4 | 79 | 111 |
|  |  | R- CACCTTGTCCCCTGGAAG | 58.4 |  |  |
| EG01_U02G010780 | Transcription factor bHLH100-like | F- CCCCAAAGGAAACAGAAACA | 56.4 | 156 | 97 |
|  |  | R- GTAGAGAGCGGAGGGAGGAG | 64.6 |  |  |
| EG01_08G007390 | Pleiotropic drug resistance protein 3-like | F- GTGGGTTTAGATGCCCTGAA | 58.4 | 159 | 100 |
|  |  | R- GTCCGATGTGATGTGCTTTG | 58.4 |  |  |
| EG01_U02G024980 | NADH dehydrogenase subunit 7 (chloroplast) | F- CGGTAAATGCACCAGAACAA | 56.4 | 91 | 96 |
|  |  | R- GCCATACGACTCAGCTCCA | 59.5 |  |  |

**Supplementary Figure S1**. General gene co-expression network for aluminum phenomena.


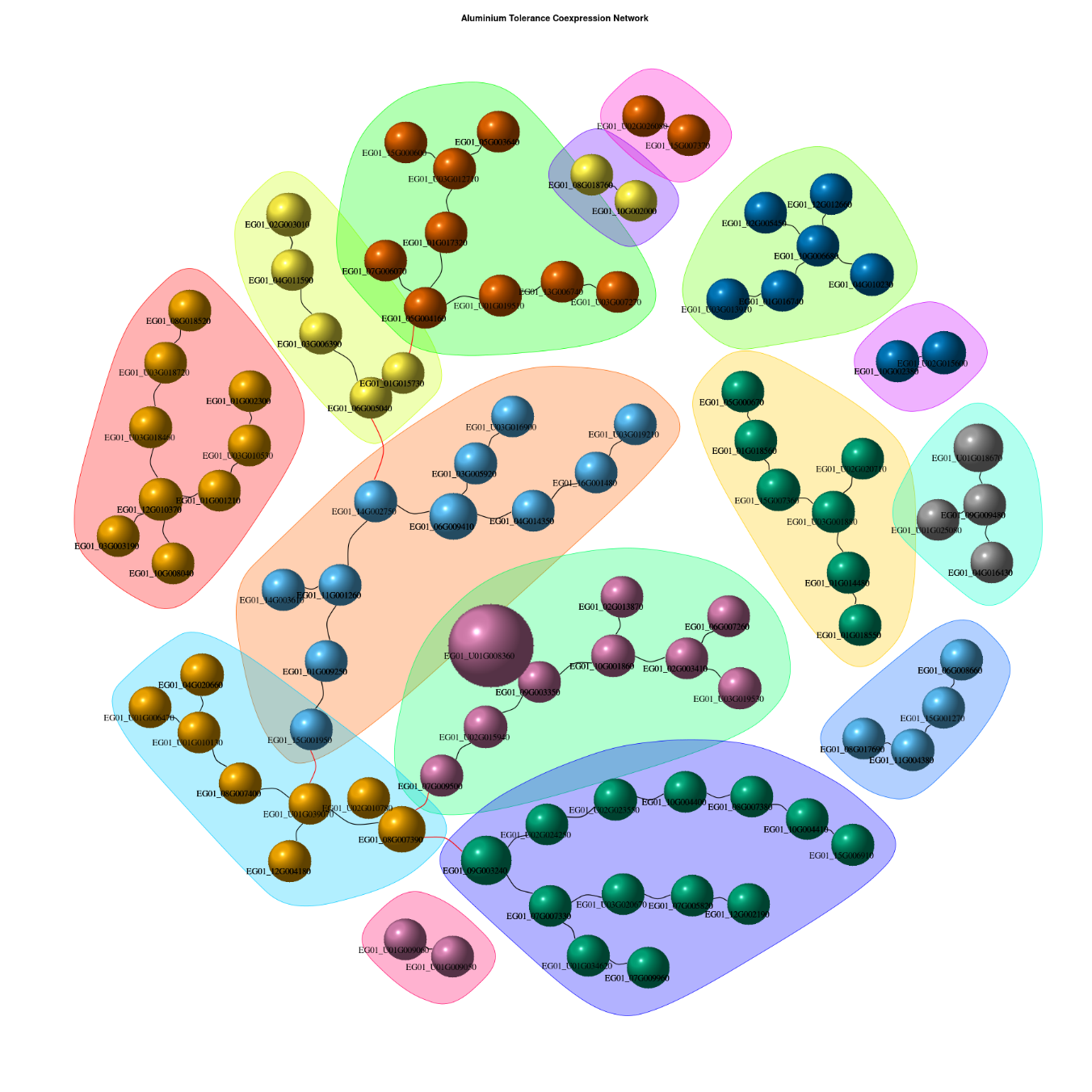


Each node (sphere or bead-like shape) represents a gene, and groups of nodes highlighted with the same color indicate a module of genes. Black edges represent a direct correlation between genes and red lines inverse correlation. Size of the nodes are proportional to the mean level expression of the gene represented by the node. The *igraph* R package was used to construct the cultivar specific co-expression networks under Al and control treatment.

**Supplementary figure S2.** Association between RNA-Seq and RT-qPCR. We found a highly association (r ) among RT-qPCR and RNA-Seq values.


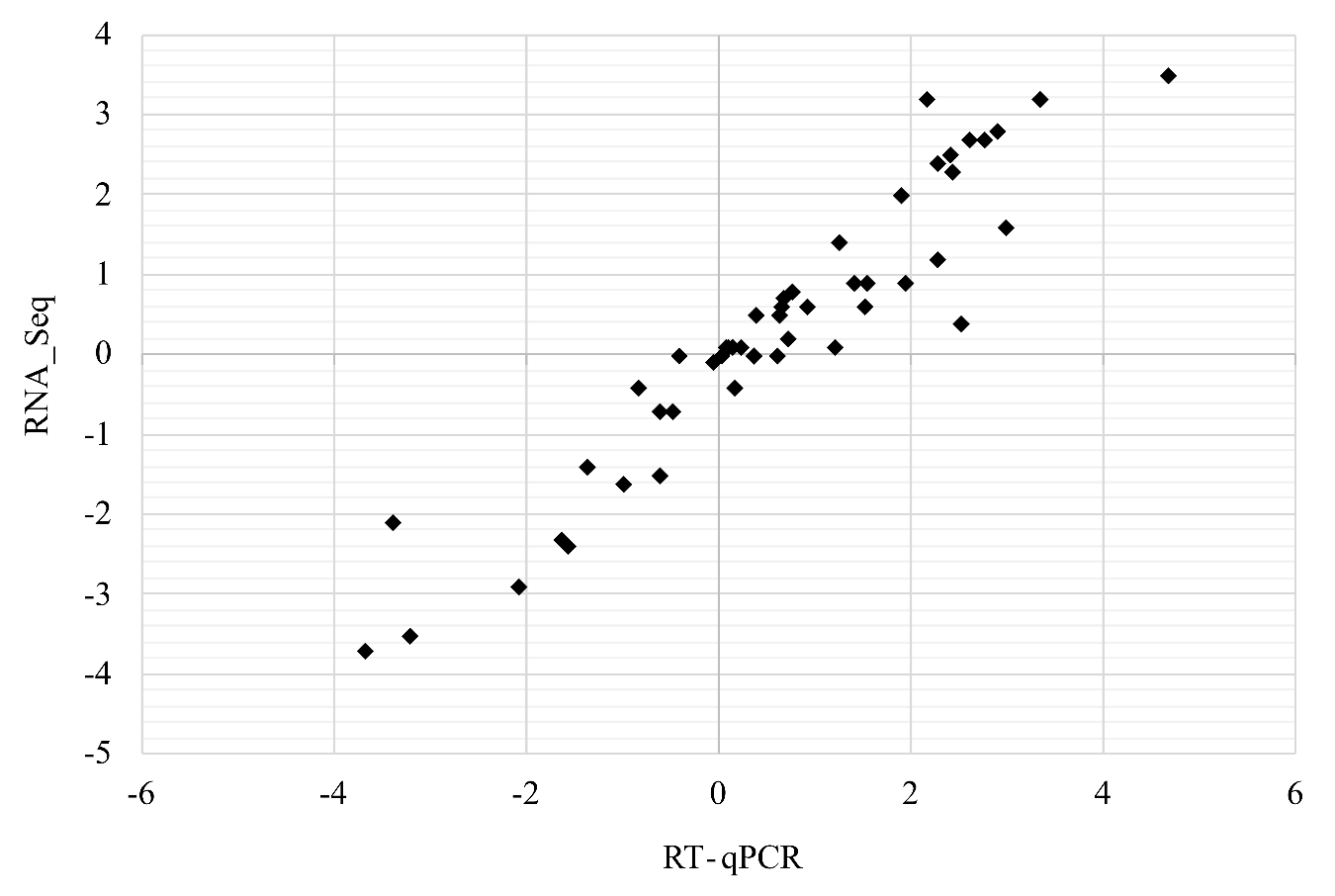

Supplement: Supplementary file 2 — Supplementary Material 2 [file 12870_2023_4354_MOESM2_ESM.docx]
